# Point-of-care Ultrasound for the Diagnosis of a “Ping Pong” Skull Fracture

**DOI:** 10.5811/cpcem.2017.12.36239

**Published:** 2018-01-11

**Authors:** Adam S. Bloom, Jonathan Auten, Joel M. Schofer

**Affiliations:** Naval Medical Center Portsmouth, Department of Emergency Medicine, Portsmouth, Virginia

## CASE PRESENTATION

**Image 1:**
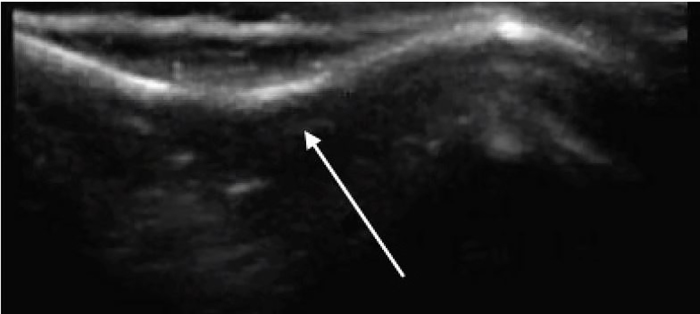
Point-of-care ultrasound demonstrating “ping pong” skull fracture (Image obtained using a liner probe, 13–6 megahertz).

**Image 2:**
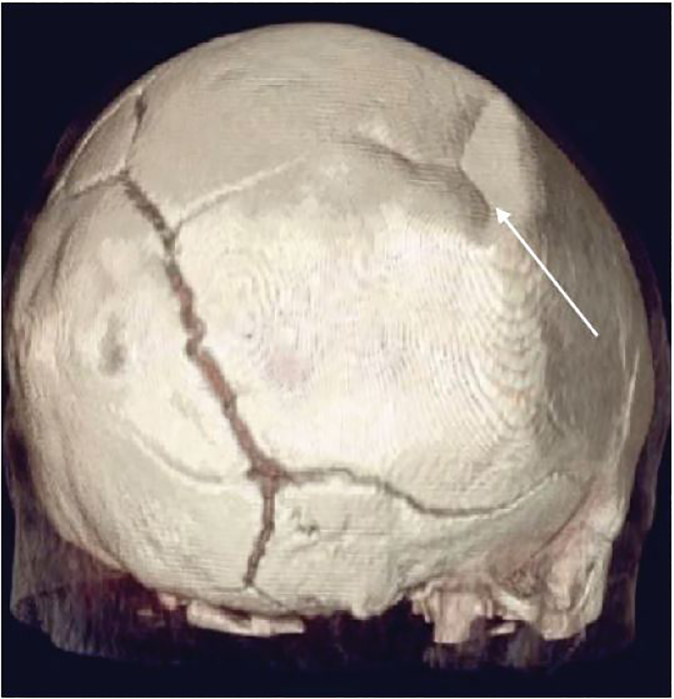
Corresponding computed tomography with three-dimensional reconstruction demonstrating “ping pong” skull fracture.

A four-month-old female presented to the emergency department after a witnessed fall from a high chair. She landed on her head but did not lose consciousness. She did not have any vomiting or altered mental status. There was a palpable defect in her right parietal skull. Point-of-care ultrasound (POCUS) demonstrated a large depression in her parietal skull consistent with a depressed skull fracture (Image 1). The fracture was confirmed by a non-contrast computed tomography (CT) of the head (Image 2). The CT was otherwise negative. The patient was admitted for observation but was discharged after an uncomplicated hospital course and was doing well at a follow-up visit.

CPC-EM Capsule

What do we already know about this clinical entity?

We know that ultrasound is a modality with excellent specificity for the diagnosis of skull fractures.

What is the major impact of the image(s)?

Ultrasound can also be used to diagnose depressed skull fractures in pediatric patients.

How might this improve emergency medicine practice?

Ultrasound may aid in the diagnosis of depressed skull fractures in pediatric patients.

## DIAGNOSIS

Depressed skull fractures in neonates are typically different from those in adults. The soft bone tends to buckle rather than break. As such, they are often referred to as “ping pong” fractures, a reference to the way a ping pong ball looks when it has been indented. They may occur as sequelae to trauma, birth, or normal uterine development.^1^ POCUS is a convenient method to quickly and accurately detect skull fractures in pediatric patients with sensitivities ranging from 88–100% in two prospective trials.^2^–^3^ While most authors agree that a CT is indicated to rule out underlying pathology once a skull fracture is identified, a consensus does not exist on the role of POCUS to safely rule out skull fractures in neonates.^4^ We believe this is the first reported case of a “ping pong” skull fracture diagnosed using POCUS.
